# Oleic Acid Protects against Hepatic Ischemia and Reperfusion Injury in Mice by Inhibiting AKT/mTOR Pathways

**DOI:** 10.1155/2019/4842592

**Published:** 2019-12-13

**Authors:** Jianrong Guo, Tao Zhang, Jian Gu, Kailin Cai, Xiuling Deng, Ke Chen, Kun Huang, Guobin Wang, Huili Li, Jiliang Wang

**Affiliations:** ^1^Department of Gastrointestinal Surgery, Union Hospital, Tongji Medical College, Huazhong University of Science and Technology, 1277 Jiefang Avenue, Wuhan, Hubei, China; ^2^Department of Endocrinology, Union Hospital, Tongji Medical College, Huazhong University of Science and Technology, 1277 Jiefang Avenue, Wuhan, Hubei, China; ^3^Institution of Cardiology, Union Hospital, Tongji Medical College, Huazhong University of Science and Technology, 1277 Jiefang Avenue, Wuhan, Hubei, China

## Abstract

Hepatic ischemia-reperfusion (I/R) injury is a serious complication in patients who have undergone hepatic surgery such as orthotopic liver transplantation and partial hepatectomy. Recently, a new cytoprotective agent, ursodeoxycholyl lysophosphatidylethanolamide (UDCA-LPE), was reported to protect against hepatic I/R injury. However, the protective mechanism of UDCA-LPE is not fully understood. Therefore, we conducted this study to explore its underlying mechanism. We used liquid chromatography-tandem mass spectrometry (LC-MS/MS) to analyze the liver lipid metabolism changes in mice during I/R. KEGG enrichment indicated that UDCA-LPE is likely to exert its protective role by regulating fatty acid (FA) metabolism. Further analysis found that UDCA-LPE significantly increased the ratio of oleic acid (OA) to palmitic acid (PA). We found that mice pretreated with OA improved tolerance to hepatic I/R injury. In addition, the phosphorylation level of AKT was markedly upregulated during oxidative stress to promote p65 nuclear translocation, triggering an inflammatory response that exacerbated cell damage and OA treatment significantly inhibited this process. Notably, OA was found to inhibit H_2_O_2_-induced oxidative stress, inflammation, and cell death in HepG2 cells. Furthermore, we found that OA supplementation to the medium did not result in a significant increase in intracellular OA, but marked increase in the ratio of OA to PA, which may be an important mechanism for the inflammatory response induced by oxidative stress during I/R. Finally, we demonstrated that OA increased the level of autophagy in HepG2 cells, which may be one of the protective mechanisms against oxidative stress. Collectively, this study revealed that FA metabolism functionally determines the oxidative stress-related inflammation caused by hepatic I/R. We hypothesize that OA treatment may be a promising strategy for preventing and treating I/R-induced liver damage.

## 1. Introduction

Hepatic ischemia-reperfusion (I/R) injury is a complication of hepatic surgery, and it can arise after liver resection and transplantation [1, 2]. Hepatic I/R injury induces oxidative stress, inflammation, and other disorders in the liver, thus leading to the liver damage in patients requiring liver surgery [3–6]. However, the mechanisms underlying the I/R injury are not completely understood. So far, only a few effective protective strategies have been discovered [7]. Ursodeoxycholyl lysophosphatidylethanolamide (UDCA-LPE), a novel anti-inflammatory agent with hepatoprotective effects, was developed by Chamulitrat et al. by coupling UDCA with a phospholipid. This drug inhibits mitochondrial damage and apoptosis, induces the survival signaling pathway, and promotes the regeneration of hepatocytes [8]. The mechanisms underlying the protective effects of this drug include shifting FA pools toward monounsaturated fatty acids (MUFA) and polyunsaturated fatty acids (PUFA), attenuating hepatofibrogenesis by impairment of TGF-*β*1/Smad2/3 signaling [9] and inherent, pronounced anti-inflammatory effects [10, 11].

Extra virgin olive oil (EVOO), a gold standard of edible oils, was reported to present a protective effect against hepatic I/R injury. EVOO combined with DHA attenuates high-fat diet- (HFD-) induced hepatic steatosis [12]. Pinto et al. [13] reported that supplementation with EVOO is associated with a reduced prevalence of NAFLD in older individuals at high cardiovascular risk. However, the molecular mechanism by which EVOO protects the liver is not completely understood. MUFAs, the main composition of EVOO (55-83%), have beneficial effects for humans. A large-scale KANWU study [14] showed that increasing relatively low levels of MUFAs in the diet and decreasing saturated FA intake could improve insulin sensitivity. In addition, it was shown that foods containing MUFAs were able to reduce low-density lipoprotein (LDL) cholesterol [15]. Other studies demonstrated that MUFAs can also increase high-density lipoprotein (HDL) [16, 17].

Oleic acid (OA, 18:1 n-9) is one of the most common MUFAs and is widely distributed and abundant in nature. People consume a large amount of OA from food, especially people who eat a Mediterranean diet [18]. In the past few decades, there has been increasing evidence showing multiple positive effects of OA on human health and disease, including modulating physiological functions, inhibiting cancer proliferation and oncogene expression, reducing inflammation, modulating leukocyte activity, lowering blood pressure, and enhancing wound healing [19]. The liver-protecting effect of EVOO might be related to the protective effects of its components such as hydroxytyrosol, OA, tocopherols, and/or PUFAs. Considering all these positive effects of OA, it is reasonable to speculate that a large part of the protective effect of EVOO on the liver is played by OA. As there is currently very little research on this topic, we conducted this study to investigate the role that MUFAs, particularly OA, play in hepatic I/R injury. We evaluated the lipid metabolism profiles of mouse livers that were subjected to a hepatic I/R injury to analyze the FA changes. In addition, a hydrogen peroxide-induced oxidative stress cell model, with and without OA pretreatment, was used to simulate hepatic I/R injury. Several observational indices were used to evaluate the protective effect of OA.

## 2. Materials and Methods

### 2.1. Ethics Statement

All animal experiments were approved by the Ethics Committee of Tongji Medical College, Huazhong University of Science and Technology, China. In addition, all the animal experiments were conducted in accordance with the National Institutes of Health (NIH) *Guide for the Care and Use of Laboratory Animals* published by the US National Institutes of Health (NIH Publication, 8th edition, 2011).

### 2.2. Animal Model

Eighty 18-week-old male C57/BL6 mice weighing 28 to 30 g were purchased from Beijing Vital River Laboratory Animal Technology Co. The animals were raised in cages under a 12/12-hour light/dark cycle at 25°C in the Animal Care Facility of Tongji Medical College.

### 2.3. Surgical Procedures

**Figure 1:**
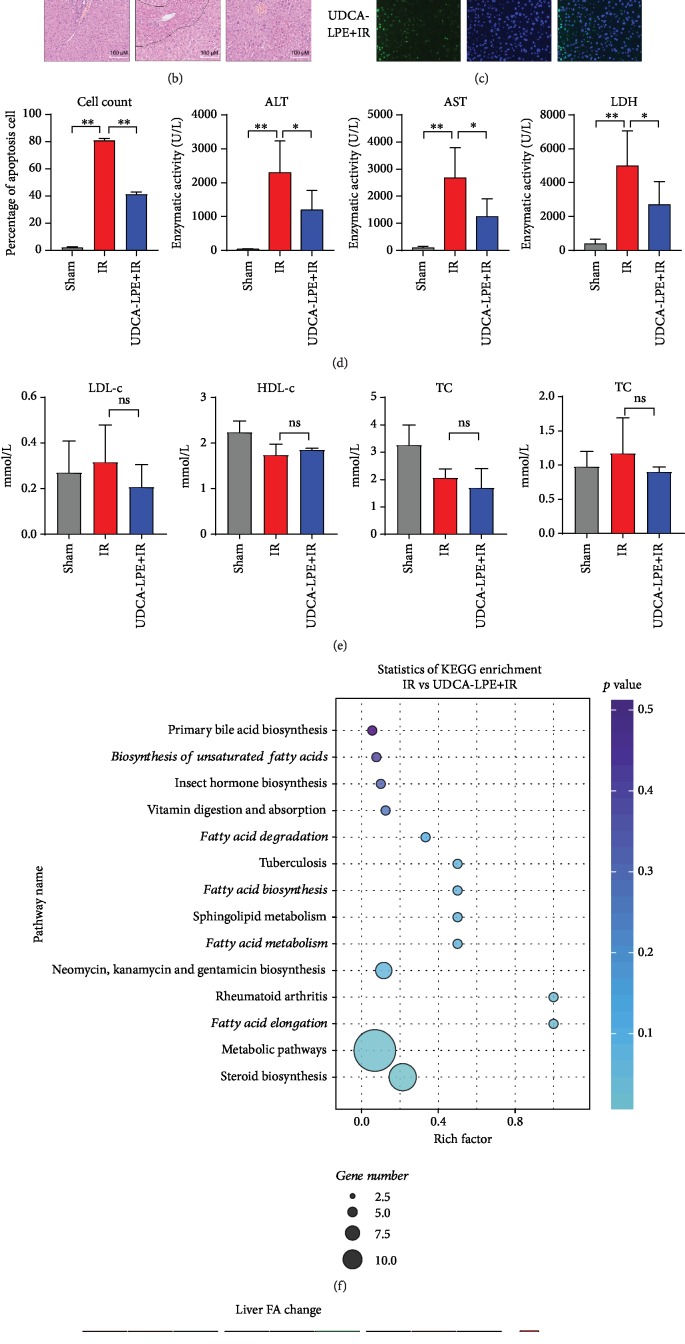
UDCA-LPE protects mice from hepatic ischemia-reperfusion injury and increased the odds ratio of OA/PA. (a) Surgical procedure. (b) Liver H&E staining after surgical procedure. (c) Mouse liver showing an apoptotic cell stained with TUNEL. (d) Liver enzymes ALT, AST, and lactate dehydrogenase were determined in the serum of mice undergoing hepatic I/R. (e) Total cholesterol (TC), triglyceride (TG), HDL-c, and LDL-c levels in the serum of each group. (f) Statistics of KEGG enrichment. (g) Heat map of liver fatty acid change determined by LC-MS. Values are expressed with mean ± SD. Each group contains three mice. ^∗∗^*p* < 0.005, ^∗^*p* < 0.05, compared with the control group. Bars indicate the standard deviation of the mean.

After a one-week adaptive phase, the animals were divided into the following three groups: sham, I/R, and UDCA-LPE+I/R. Each group contained at least six mice. The surgical procedures were performed as previously described to induce hepatic I/R injury implicating 70% of the liver [20]. As shown in Figure 1(a), fasted mice were anesthetized with pentobarbital sodium (50 mg/kg) by an intraperitoneal injection and underwent a midline incision to expose the liver. An atraumatic clamp was placed across a branch of the portal triad to block the blood supply to the median and left lateral liver lobes to induce ischemia for 90 min. Following unclamping of the liver, hepatic reperfusion was allowed for 2 h, and this procedure represented the I/R group. UDCA-LPE stock was prepared at 5 mg/mL in 0.5% CMC. In the I/R group, the vehicle CMC was injected intraperitoneally. In the UDCA-LPE+I/R group, two doses of 50 mg/kg UDCA-LPE were injected intraperitoneally at 30 min prior to clamping and just prior to reperfusion (Figure 1(a)). The sham groups only received a switching abdominal surgery. After surgery, blood samples were collected and centrifuged at 3500 r/min for 15 min. Livers were harvested and fixed. Parts of the liver samples were sectioned into 1 mm slices, then stained with haematoxylin and eosin, photographed using a NanoZoomer S360 (Hamamatsu, Japan), and analyzed using NZAcquire software. The rest of the samples were stored at -80°C for subsequent tests.

### 2.4. Serum Transaminase

Serum transaminases and triglyceride were detected by Aeroset-2000 automatic biochemical analyzer (Instrument Laboratory, USA).

### 2.5. Cell Lines and Oxidative Cell Model

The human hepatocellular carcinoma cell line HepG2 was purchased from the American Type Culture Collection (ATCC, USA). Cells were cultured in DMEM (Gibco, USA) with 10% FBS (ScienCell, USA) and kept in a humidified atmosphere at 37°C with 5% CO_2_ in an incubator (Thermo Fisher Scientific Inc., USA). To generate the oxidative model, we treated the cells with various concentrations of H_2_O_2_ for 3 h. To render the OA mother liquor more soluble, we dissolve the OA into 1% DMSO and subjected it to ultrasonification for 10 min before use. To generate the oxidative model, we treated the cells with various concentrations of H_2_O_2_ for 3 h.

### 2.6. Proliferation Assay

The CCK-8 (Boster Biological Technology, Ltd., China) proliferation assay was performed according to the manufacturer's instructions for the indicated time.

### 2.7. LC-MS/MS Analysis

#### 2.7.1. Lipid Extraction

Tissue sample was grounded by liquid nitrogen. Then, tissue samples were firstly bath sonicated for 2 min with 400 *μ*L ice-cold 75% to break up the cells. Next, 1 mL MTBE was added and the samples were shaken for 1 h at room temperature. Next, phase separation was induced by adding 250 *μ*L water, letting it sit for 10 min at room temperature and centrifuging for 15 min at 14,000g, 4°C. Because of the low density and high hydrophobicity of MTBE, lipids and lipophilic metabolites are mainly extracted to the upper MTBE-rich phase. The lipid was transferred to fresh tubes and dried with air nitrogen.

#### 2.7.2. UPLC-MS Mobile Phases

Lipid analysis was performed on a Q Exactive orbitrap mass spectrometer (Thermo, CA). Mobile phase A is prepared by dissolving 0.77 g of ammonium acetate to 400 mL of HPLC-grade water, followed by adding 600 mL of HPLC-grade acetonitrile. Mobile phase B is prepared by mixing 100 mL of acetonitrile with 900 mL isopropanol.

#### 2.7.3. Lipid Analysis

Lipids were identified and quantified using LipidSearch 4.1.30 (Thermo, CA). Mass tolerance of 5 ppm and 10 ppm was applied for precursor and product ions. Retention time shift of 0.25 min was performed in “alignment.” M-score and chromatographic areas were used to reduce false positives.

### 2.8. Detection of Apoptosis

HepG2 cells were seeded at a density of 2 × 10^6^ cells/mL of the DMEM medium with 10% FBS on 6-well plates to a final volume of 2 mL. After treatment with OA and H_2_O_2_, the cells were collected, washed twice with PBS, and then suspended in 1x Binding Buffer at a concentration of 1 × 10^6^ cells/mL. The cells were subjected to 5 *μ*L of FITC-Annexin V and 5 *μ*L propidium iodide (PI) staining using the Annexin V-FITC apoptosis kit (KeyGEN BioTECH, China). Next, 100 *μ*L of the solution was transferred to a 5 mL culture tube and incubated for 30 min at RT (25°C) in the dark. The apoptosis ratio was quantified using flow cytometry. Data were presented by the system software (Cell Quest; BD Biosciences).

### 2.9. Detection of ROS, GSH, and MDA

To detect the production of ROS, GSH, and MDA, HepG2 cells were seeded at a density of 2 × 10^6^ cells/mL of the DMEM with 10% FBS on 6-well plates. Reactive Oxygen Species Assay Kit, GSH and GSSG Assay Kit, and MDA Assay Kit (Beyotime Biotechnology, China) were used following the manufacturer's instructions.

### 2.10. Western Blotting

Cells and tissues were lysed using RIPA lysis buffer supplemented with 1% PMSF and 1% phosphorylase inhibitor. The protein concentration was determined using either the BCA or Bradford protein assay kit (Beyotime Biotechnology, China). Boiled lysates were subjected to SDS-PAGE and transferred to a polyvinylidene fluoride membrane (Millipore, Billerica, MA, USA), which was blocked with 5% BSA blocking buffer for 1 h and subsequently incubated with the indicated primary antibodies overnight at 4°C. Following incubation with HRP-conjugated secondary antibodies (diluted 1 : 5000) for 1 h at room temperature, the membranes were treated with ECL reagents (Meilunbio, China). Autoradiograms were scanned, and the labelled bands were quantified using Image-Pro Plus software. The following primary antibodies and dilutions were used: anti-GAPDH (#5174, diluted 1 : 1000), anti-p-PI3K (#4228, diluted 1 : 1000), anti-p-AKT (#4060, diluted 1 : 500), anti-caspase 3 (#9662, diluted 1 : 500), anti-LC3A/B (#4108, diluted 1 : 1000), and anti-p65 (#8242, diluted 1 : 1000) were purchased form Cell Signaling Technology (CST, USA); anti-renalase (GTX89570, diluted 1 : 1000) was purchased from GeneTex (Irvine, CA, USA). Anti-AKT (10176-2-AP, diluted 1 : 3000), anti-mTOR (20657-1-AP, diluted 1 : 500), anti-Bcl2 (12789-1-AP, diluted 1 : 1000), and anti-PCNA (10205-2-AP, diluted 1 : 1000) were purchased form Proteintech Group (Proteintech, China). Anti-p-mTOR (ab109268, diluted 1 : 1000) was purchased from Abcam (CA, USA).

### 2.11. Gene Expression Analysis by Quantitative Real-Time PCR

RNA was extracted from liver tissues and cells tissues using RNAiso Reagent (TaKaRa, China). cDNA was synthesized using the HiScript II Q RT SuperMix for qPCR (+gDNA wiper) (Vazyme Biotech Co., Ltd.) according to the manufacturer's instructions. PCR reactions were prepared using the ChamQTM SYBR qPCR Master Mix (Vazyme Biotech Co., Ltd.) and performed using a StepOnePlus™ System (Thermo Fisher Scientific Inc., USA). The sense and antisense primers are shown in Tables 1 and 2.

### 2.12. Regents

The synthesis of UDCA-LPE was reported previously [20]. For UDCA-LPE used in this study, the same synthesis procedure was performed by ChemCon (Freiburg, Germany), donated by Prof. Walee Chamulitrat (Department of Internal Medicine IV, University Heidelberg Hospital, Heidelberg, Germany). Recombinant Human IGF-I Protein was purchased from R&D Systems (CA, USA). Oleic acid was purchased from Sigma-Aldrich (CA, USA). H_2_O_2_ was purchased from Sinopharm Chemical Reagent Co., Ltd (Shanghai, China).

### 2.13. Data Analysis

The fluorescent density was analyzed with Image Pro Plus software. Densitometric analysis of Western blot was carried out with ImageJ software. Data are presented as means ± SD. Statistical analysis was performed using GraphPad PRISM. All data sets were tested for normality of distribution using the Shapiro-Wilk test. Comparison between two groups was assessed using two-tailed unpaired Student's *t*-test or paired Student's *t*-test. One-way/two-way ANOVA was used to perform the statistical analysis among more than two groups, followed by Tukey's post hoc test for multiple-group comparisons. A *p* value < 0.05 was considered statistically significant.

## 3. Results

### 3.1. UDCA-LPE Protects Mice from Hepatic Ischemia-Reperfusion Injury

Our previous study [20] has proved that UDCA-LPE was able to protect mice from liver I/R injury. However, we did not study the role of lipid metabolism in it before. Therefore, we repeated this experiment and confirmed the protective effect of UDCA-LPE. As described in Materials and Methods, our experimental design is depicted in Figure 1(a). The histological evaluation revealed that I/R caused massive hepatic necrosis. A significant improvement was observed in the UDCA-LPE+I/R group (Figure 1(b)) which is consistent with the observed necrosis during I/R showing in the TUNEL staining (Figure 1(c)). I/R increased the levels of serum alanine transaminase (ALT), aspartate transaminase (AST), and lactate dehydrogenase (Figure 1(d)). UDCA-LPE administration in mice undergoing I/R significantly inhibited the elevation with stronger effects in the UDCA-LPE+I/R group (Figure 1(d)).

### 3.2. UDCA-LPE Increases the Odds Ratio of Oleic Acid to Palmitic Acid in the Liver of Mice

UDCA-LPE did not induce a significant change in lipid metabolism in mice during I/R injury. The TC, HDL-c, LDL-c, and TG levels in serum of mice did not seem to change significantly after treatment with UDCA-LPE before I/R injury (Figure 1(e)). Hence, we conducted lipidomic analysis of total mouse hepatic FA composition to understand the effects of lipid metabolism on the liver during I/R injury. KEGG enrichment (Figure 1(f)) analysis indicated that UDCA-LPE was most likely to participate in FA metabolism pathways during I/R injury. The heat map of changes in liver fatty acids in Figure 1(g) shows that UDCA-LPE can significantly increase the odds ratio of OA to PA.

### 3.3. Oleic Acid Alleviates Mouse Hepatic Ischemia-Reperfusion Injury

**Figure 2:**
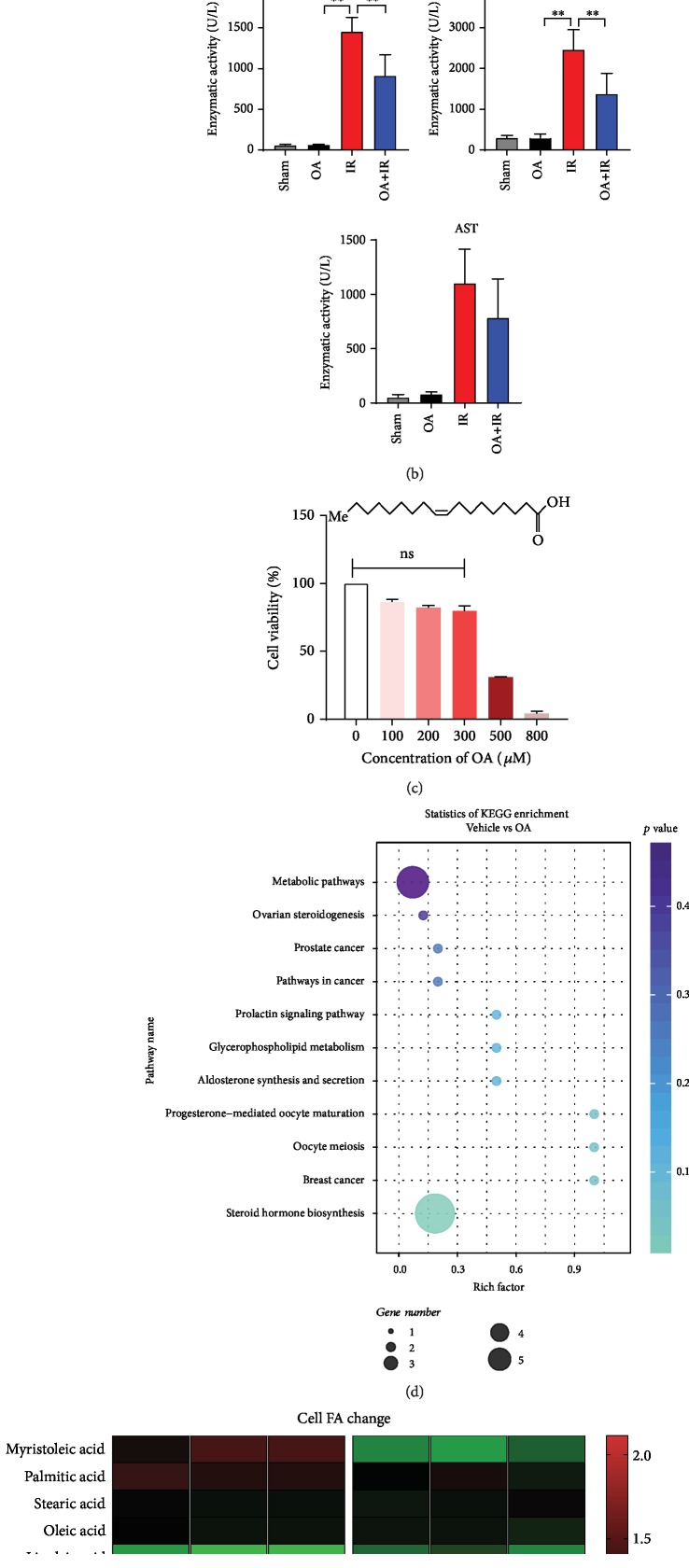
Oleic acid alleviates mouse hepatic ischemia-reperfusion injury. OA group mice were pretreated with 2 weeks of intragastric administration of oleic acid (250 mg/kg) prior to surgery. (a) Liver H&E staining after surgical procedure. (b) Serum ALT, AST, and LDH levels in each group. (c) Linear molecular structure of oleic acid; HepG2 cells were treated with various concentrations of OA for 24 h, and then, cell viability was measured by CCK-8 assay; 100 *μ*M OA has no significant effect on cell viability and was used in all subsequent experiments. (d) Statistics of KEGG enrichment. (e) Heat map of cell fatty acid change determined by LC-MS. Values are expressed as the mean ± SD; ^∗∗^*p* < 0.01, ^∗^*p* < 0.05. Bars indicate the standard deviation of the mean.

To validate our hypothesis, we conducted an *in vivo* experiment. Adult male C57 mice were randomly divided into three groups. The sham group only received switching abdominal surgery. The I/R group underwent surgical procedures as previously described, while the OA+I/R group mice received OA intragastric administration (250 mg/kg) for two weeks before undergoing the same surgical procedures. By comparing H&E staining of mouse liver sections, we found that the OA+I/R group had less liver damage than the I/R group. Further biochemical tests were consistent with H&E staining of liver sections (Figure 2(a)). OA can reduce the level of liver enzymes during liver I/R in mice, especially ALT and LDH levels (Figure 2(b), *p* < 0.05).

To investigate the effect of OA on cell lipid metabolism, HepG2 cells were treated with 100 *μ*M OA for 24 h. The FA composition in HepG2 cells was analyzed by LC-MS/MS as described before. FA changes are displayed in the heat map (Figure 2(e)). KEGG enrichment analysis (Figure 2(d)) indicated the pathways that OA is most likely to be involved in. It is worth noting that supplementation of OA did not significantly increase intracellular OA content, but increased the odds ratio of OA/PA (Figure 2(e)).

### 3.4. Oleic Acid Protects Hepatic Cells from H_2_O_2_-Induced Inflammation

**Figure 3:**
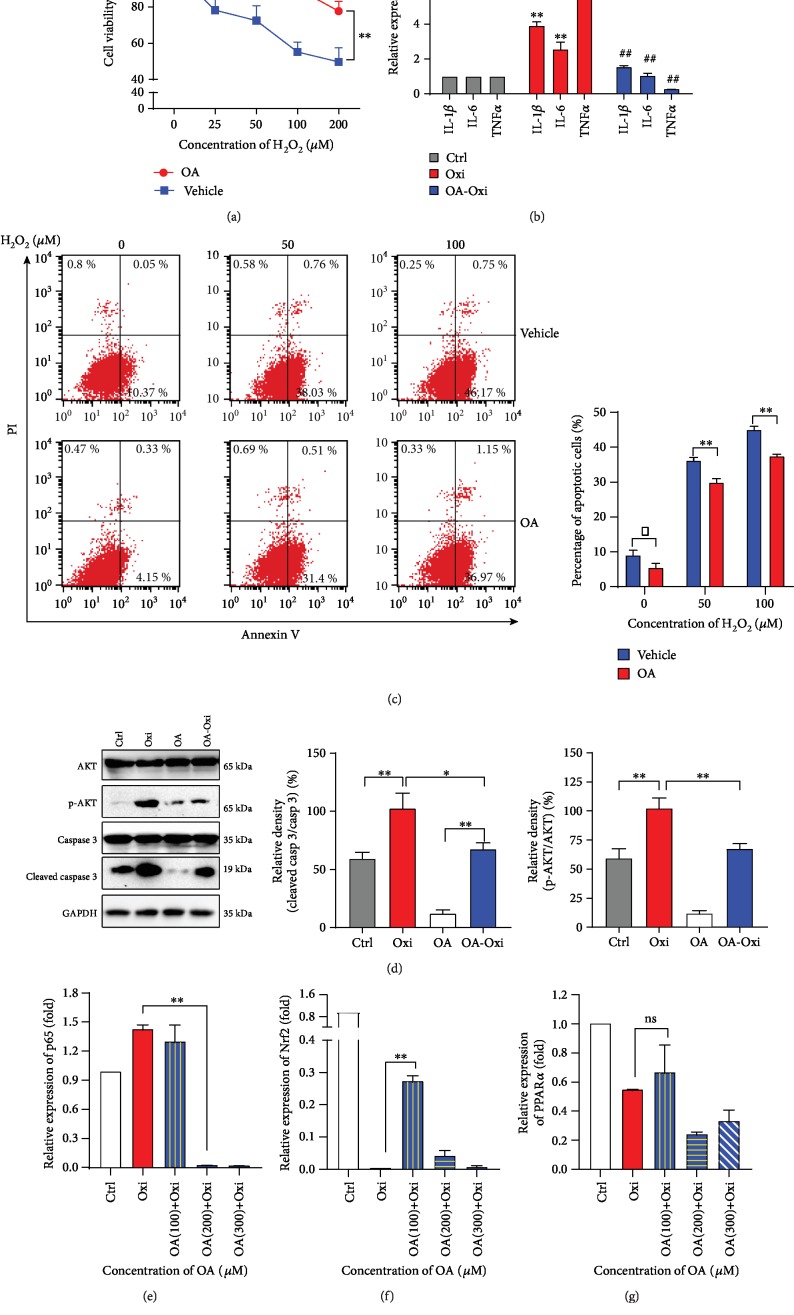
Oleic acid protects hepatic cells from H_2_O_2_-induced inflammation. HepG2 cells were treated with various concentrations of H_2_O_2_ for 3 h with or without OA pretreatment. (a) Cell viability was measured by CCK-8 assay. (b) Relative liver mRNA expression of TNF-*α*, IL-1*β*, and IL-6; the OA-Oxi group received a 100 *μ*M OA pretreatment for 24 h. (c) Percentage of apoptotic cells. Western blot analysis of AKT/p-AKT, caspase 3/cleaved caspase 3 protein levels after oxidative stress in HepG2 cells with or without OA treatment. (e–g) Relative liver mRNA expression of p65, Nrf2, and PPAR*α*; ^∗∗^*p* < 0.01, ^∗^*p* < 0.05. Data are plotted as the mean ± SD from three independent experiments. Bars indicate the standard deviation of the mean.

To mimic I/R injury in cell model, HepG2 cells were selected to undergo various concentrations of H_2_O_2_ treatment. As a result, 100 *μ*M H_2_O_2_ was considered the best stimulation concentration (Fig. [Supplementary-material supplementary-material-1]). When pretreated with OA, the destructive effects of H_2_O_2_ on cells were reduced. The cell viability in the OA group was higher than that in the vehicle group (Figure 3(a)). Consistently, results of FACS analysis (Figure 3(c)) demonstrated that as the concentration of H_2_O_2_ increases, the number of apoptotic cells increases. However, it is worth noting that the OA group had lower number of apoptotic cells compared to the vehicle group. Western blot results also supported this observation. Cleaved caspase 3 level was markedly lower with OA treatment compared to groups without OA treatment (Figure 3(d), *p* < 0.05).

HepG2 cells were pretreated with different concentrations of OA before undergoing oxidative stress. The expression of inflammation factors, such as TNF-*α*, IL-*β*, and IL-6, increased after H_2_O_2_ treatment, which was consistent with the expression of inflammation factors after hepatic I/R injury (unpublished observations). However, inflammation factor expression in the OA+Oxi (100 *μ*M) group sharply decreased compared with that in the Oxi group (Figure 3(b), *p* < 0.01). Western blot analysis identified that the PI3K/AKT/mTOR pathway was activated ([Supplementary-material supplementary-material-1]) under oxidative stress. The mRNA level of p65 expression showed the opposite tendency, which was consistent with the expression level of the inflammatory cytokines (Figure 3(e)). The mRNA expression of nuclear factor erythroid 2-related factor 2 (Nrf2) sharply decreased under oxidative stress (Figure 3(f)). However, OA does not seem to reverse the downregulation of peroxisome proliferator-activated receptor *α* (PPAR*α*) caused by oxidative stress (Figure 3(g)).

### 3.5. Oleic Acid Protects Hepatic Cells via Alleviating H_2_O_2_-Induced Oxidative Stress

**Figure 4:**
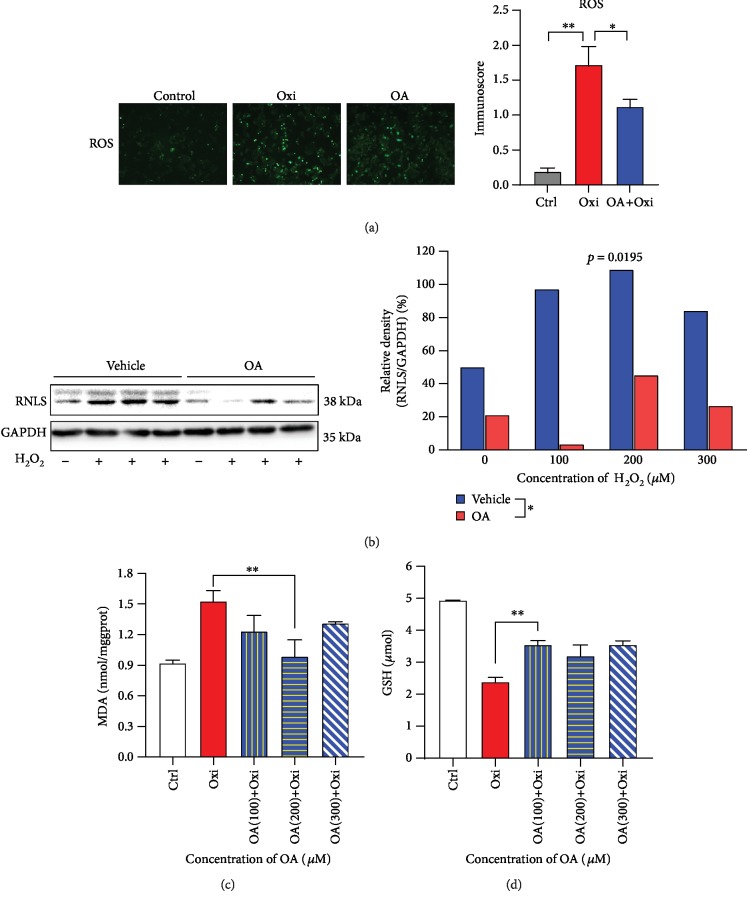
Oleic acid protects hepatic cells via alleviating H_2_O_2_-induced oxidative stress. (a) ROS fluorescence and its corresponding fluorescence intensity score. (b) Western blot analysis of renalase protein levels. (c, d) The intracellular endogenous MDA and GSH in HepG2 cells; ^∗∗^*p* < 0.01, ^∗^*p* < 0.05. Data are plotted as the mean ± SD from three independent experiments. Bars indicate the standard deviation of the mean.

ROS fluorescence and immunoscore showed that OA was able to reduce the intercellular ROS production in HepG2 cells after H_2_O_2_ treatment (Figure 4(a), *p* < 0.05). The MDA level increased during oxidation; however, the OA treatment (200 *μ*M) significantly lowered the MDA level caused by H_2_O_2_ (Figure 4(c), *p* < 0.05). The total GSH in HepG2 cells was measured. After H_2_O_2_ treatment, the GSH level decreased, while the degree of decline was significantly lower in the OA (100 *μ*M)+Oxi group (Figure 4(d), *p* < 0.05). Western blot analysis demonstrated that the protein expression level of renalase was elevated during oxidation. In the OA-treated group, the degree of increase was not as high as that compared with the vehicle group (Figure 4(b)).

### 3.6. OA Inhibits the Nuclear Translocation of p65, Resists Apoptosis, and Enhances Autophagy during Oxidative Stress in HepG2 Cells

**Figure 5:**
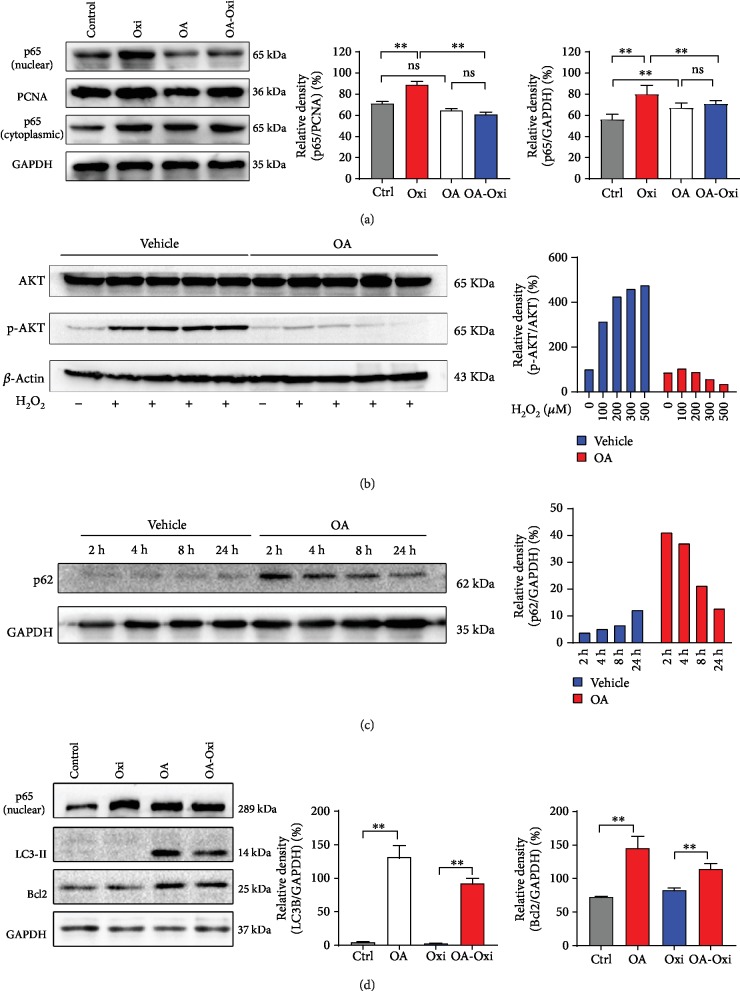
Oleic acid inhibits the nuclear translocation of p65, resists apoptosis, and enhances autophagy during oxidative stress in HepG2 cells. (a, b, d) Western blot analysis of cytoplasm and nuclear p65, AKT/p-AKT, p-mTOR, LC3, and Bcl2 protein levels after oxidative stress in HepG2 cells with or without OA treatment. (c) HepG2 cell were treated with 100 *μ*M OA for 2 h, 4 h, 12 h, and 24 h. Western blot analysis of p62 protein levels. Values are expressed as the mean ± SD from three independent experiments; ^∗∗^*p* < 0.01, ^∗^*p* < 0.05, compared with the control group. ns means no significance. Bars indicate the standard deviation of the mean.

Western blot analysis of the cytoplasm and nuclear p65 protein demonstrated that there was no significant change in cytoplasmic p65 protein levels in HepG2 cells, whether stimulated with H_2_O_2_ or treated with OA (Figure 5(a)). However, we found a significant change in the level of p65 in the nucleus (Figure 5(a), *p* < 0.01). Oxidative stress increases the level of p65 in the nucleus, both in the control group and in the OA group. Western blot results indicated that OA markedly decreased the phosphorylation level of AKT (Ser473) in the presence of H_2_O_2_. In the vehicle group, H_2_O_2_ significantly increased the phosphorylation level of AKT (Figures 3(d) and 5(b)). p62 was activated upon OA treatment (2 h); however, the protein level of p62 decreased with treatment time compared with vehicle group (Figure 5(c)). Significantly increased levels of Bcl2 and LC3-II proteins were observed in the OA group compared with the control group when H_2_O_2_ was not present. After H_2_O_2_ treatment, the Bcl2 and LC3-II levels remained unchanged in the vehicle group, while they were sharply elevated in the OA group (Figure 5(d), *p* < 0.01).

### 3.7. AKT Phosphorylation Activator Partially Reverses OA's Effects

**Figure 6:**
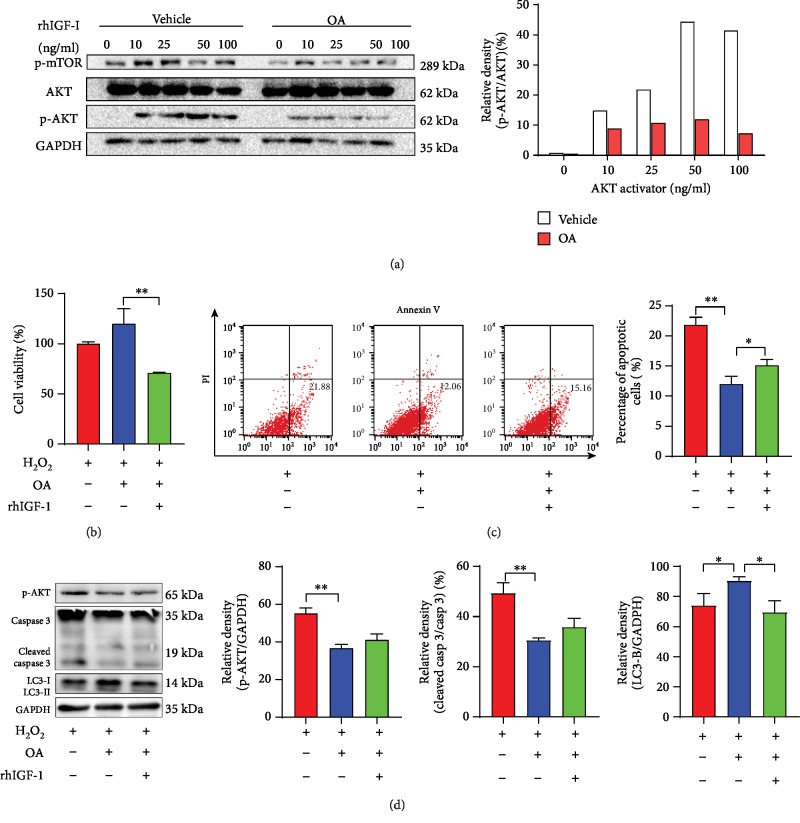
AKT phosphorylation activator partially reverses OA effects. (a) Western blot analysis of p-mTOR and AKT/p-AKT protein levels with various concentrations of rhIGF-I pretreatment. HepG2 cells were pretreated with/without 50 ng/mg rhIGF-I, for 30 min before oxidation, apoptosis cells were detected with Annexin V-FITC kit using FASC, and cell viability was measured with CCK-8 assay. (b) Cell viability of HepG2 cells. (c) Percentage of apoptotic cells. (d) Western blot analysis of p-AKT, caspase 3/cleaved caspase 3, and LC3 protein levels in HepG2 cells. Values are expressed as the mean ± SD from three independent experiments; ^∗∗^*p* < 0.01, ^∗^*p* < 0.05. Bars indicate the standard deviation of the mean.

For better understanding of the inhibitory action of OA on the AKT/mTOR signaling pathway, we used recombinant human IGF-I (10 *μ*M, 25 *μ*M, 50 *μ*M, and 100 *μ*M), a frequently used AKT activator, to treat HepG2 cells for 30 min. As expected, the p-AKT level increased with increase in concentration of rhIGF-I and reached its peak at 50 *μ*M rhIGF-I (Figure 6(a)). Similarly, the p-AKT level increased in parallel with the increase in concentration of rhIGF-I in the OA-treated group. However, the degree of upregulation in the OA-treated group was noticeably lower than that in the control group (Figure 6(a), *p* < 0.01). The results of CCK-8 assay demonstrated that the AKT activator can reverse the protective effect of OA under oxidative stress in HepG2 cells (Figure 6(b), *p* < 0.01). After pretreatment with 50 *μ*M rhIGF-I, cells were treated with 50 *μ*M H_2_O_2_ for 3 h. The FACS results (Figure 6(c)) showed that when subjected to oxidative stress, rhIGF-I pretreatment slightly increased the number of apoptotic cells compared with those without rhIGF-I pretreatment. The level of cleaved caspase 3 showed the same tendency (Figure 6(d)), though without statistical significance. However, AKT activator successfully downregulated level of LC3-B increased by OA (Figure 6(d), *p* < 0.05).

## 4. Discussion

Our experiments demonstrated that UDCA-LPE protects the liver from I/R injury in mice, which is consistent with a previous study [20]. We used lipid metabolomics combined with bioinformatics techniques to demonstrate that the protective mechanism of UDCA-LPE is closely related to FA metabolism. However, our results indicated that UDCA-LPE has no significant effect on lipid metabolism in serum, but can significantly alter the lipid composition of the liver. Our study also identified that UDCA-LPE can significantly increase the ratio of OA/PA in the liver. This may be one of the important mechanisms of protective effect of UDCA-LPE on the liver against I/R injury. In agreement with this view, OA supplementation was shown to attenuate liver I/R injury *in vivo*, since PA is generally considered harmful to the liver [20–22]. In the cell model, relatively low concentration of OA supplementation was shown to protect HepG2 cells from H_2_O_2_-induced injury. LC-MS/MS results showed that OA supplementation does not significantly increase intracellular OA content, but can increase the OA/PA ratio. H_2_O_2_ was used to mimic I/R injury, which has been widely used to research I/R [23, 24]. Our results indicated that OA supplementation can reduce the damage of H_2_O_2_ on cells and reduce the number of apoptotic cells.

A large body of research [25–27] has proved that inflammation and oxidative stress play an important, adverse role in hepatic I/R injury. Recently, studies reported that supplementation with EVOO prevents oxidative stress [28, 29]. As OA is one of the main components of EVOO, we explored the association between OA and EVOO. According to our findings, OA was able to decrease hepatic expression of TNF-*α*, IL-1*β*, IL-6, and p65 to levels comparable to the control group. As reported, liver inflammation induced by oxidative stress is closely related to the activity of Nrf2 and PPAR*α*. Ligand activation of PPAR*α* induces antioxidant, metabolic, and anti-inflammatory responses. Our study proved that OA supplementation only upregulates Nrf2 mRNA expression during oxidative stress. OA also tends to reduce the mRNA expression level of PPAR*α*.

The mechanisms of I/R injury are diverse, but the emergence of ROS is one of the most critical factors [30–32]. The sources of ROS are xanthine oxidase, NADPH oxidase (Nox), mitochondria, and uncoupled nitric oxide synthase and have become the current priority targets for therapeutic intervention against reperfusion-induced organ dysfunction and tissue damage [30, 33]. ROS leads to cell death by mediating apoptosis, mitoptosis, necrosis, and necroptosis [32]. In our study, we found that OA can significantly reduce the amount of intracellular ROS produced during oxidative stress. This clearance effect is partly due to the enhancement of intracellular glutathione (GSH) production induced by OA ([Supplementary-material supplementary-material-1]). Malondialdehyde (MDA), produced from PUFAs by both chemical reactions and reactions catalyzed by enzymes, represents the intracellular lipid peroxidation level [34]. Our study also confirmed that OA was able to limit intracellular lipid peroxidation levels induced by H_2_O_2_. Our previous study found that renalase is highly sensitive and responsive to oxidative stress *in vitro* and *in vivo* [35]. In this study, we found OA decreased the expression of renalase induced by H_2_O_2_. Previous studies have confirmed that OA is effective in the prevention of diverse types of digestive disorders such as inflammatory bowel disease and cardiovascular disease due to its antioxidant capacity [36–38]. The antioxidant effect of OA in liver I/R injury is consistent with the existing literature.

The nuclear transcription of NF-*κ*B is activated via AKT signaling pathway under oxidative stress [39], which is independent of PI3K phosphorylation. The activation of these signaling pathways leads to the production of various proinflammatory mediators, such as TNF-*α*, IL-1*β*, IL-6, induced nitric oxide synthase (iNOS), and cyclooxygense-2 (COX-2), which are capable of amplifying the process of inflammation [40]. NF-*κ*B is a highly inducible transcription factor that plays an important role in the hepatic acute phase response, innate/adaptive immunity, and cellular survival through the induction of genetic networks [41, 42]. RELA, also known as p65, is a REL-associated protein involved in NF-*κ*B heterodimer formation, nuclear translocation, and activation. In addition, phosphorylation of AKT (Ser473) is associated with p65 [43]. We identified an increase in nuclear protein level of p65 under oxidative stress conditions, and OA significantly inhibited this process. It is worth mentioning that in the cytoplasm, we did not detect changes in protein expression level of p65. However, at the mRNA level, we found that OA can significantly reduce the mRNA transcription level of p65. This may be due to the certain time lag between protein expression and mRNA expression. Combining previous reports with our research results, we hypothesize that OA reduces nuclear transcription of p65 by inhibiting phosphorylation of AKT, thereby reducing inflammatory factor expression. Additionally, oxidative stress-induced ROS and inflammatory factors lead to apoptosis, which is highly associated with AKT phosphorylation. OA is able to clear ROS and downregulate the expression of inflammation factors by blocking AKT phosphorylation in an unknown manner, thus protecting cells from apoptosis.

Autophagy is a biological process in which macromolecules and damaged organelles in the cytoplasm are degraded [32]. It is a self-degrading system that keeps normal cells in a homeostatic environment. Autophagy-associated cell death is considered an important mechanism for nonapoptotic cell death [44]. However, the role of autophagy in liver I/R injury remains controversial. Moderate autophagy levels are beneficial for hepatocytes to maintain homeostasis, but enhanced autophagy may further aggravate the damage [45, 46]. In either case, autophagy is closely related to liver I/R injury. Autophagy is regulated by autophagy-related genes (ATG) and can be induced by a variety of factors. LC3-II is produced during autophagy and thus can be used as an autophagosomal marker [47, 48]. The insulin-like growth factor I- (IGF-I-) AKT-mTOR pathway (IIS) is involved in multiple bioprocesses like aging, longevity, and cell survival/death signaling [49]. IIS integrates a wide array of metabolic signals, cross-talk with p53, NF-*κ*B, or ROS, and influences gene expression to shape the cellular metabolic profile and stress resistance [49–53]. mTOR is located downstream of PI3K-AKT signaling, regulates cell growth, and inhibits the initial process of autophagy [45, 46, 54]. In this study, we identified for the first time that OA reduces the expression of p-mTOR protein by inhibiting the phosphorylation of AKT, thereby abolishing the inhibition of autophagy by mTORC1 and thus promoting autophagy. This finding is consistent with the findings of Qin et al. and provides a rationale for a novel therapeutic strategy for managing liver I/R injury [54].

The mechanism of OA inhibiting the phosphorylation of AKT remains unclear. Activation of PI3K by extracellular stimuli results in activation of AKT in almost all cells and tissues. PI3K and its lipid products are generally considered to be obligate and rate limiting for proper AKT activation [55]. However, our study demonstrated that OA blocks the AKT phosphorylation in a PI3K-independent manner. This may be due to the increase in p-PI3K level when OA was introduced (Fig. [Supplementary-material supplementary-material-1]), while p-AKT level was suppressed. The phosphorylation level of AKT is positively correlated with the number of PIP3, which is mainly phosphorylated by PIP_2_ in the cell and nuclear membranes [55, 56]. The molecular structure of OA has great similarity with the lipid part of PI-4,5-P_2_ and PI-3,4,5-P_3_, which means it is likely to competitively combine with AKT, thereby inhibiting its phosphorylation. We will explore its mechanism in depth in a subsequent study.

## 5. Conclusions

In this study, we found that UDCA-LPE can significantly increase the ratio of OA/PA in the liver, which may account for its protective effect against the liver I/R injury. OA supplementation demonstrated the alleviation of mouse liver I/R injury. A relatively low dose of OA protects against oxidative stress, inflammation, and apoptosis and enhances autophagy in HepG2 cells. Furthermore, OA supplementation reversed I/R-induced hepatocyte death. As a MUFA, OA can eliminate ROS and MDA produced during hepatic I/R injury and suppress the expression of inflammatory factors by inhibiting nuclear transcription of p65 by suppressing AKT phosphorylation. In addition, OA induces the enhancement of autophagy by suppression of AKT/mTOR pathways, thus protecting cells from oxidative stress. Considering all these positive effects, OA supplementation represents a potential suitable therapeutic strategy preventing liver I/R injury. It is reasonable to expect that there will continue to be many important mechanistic and medical insights regarding lipid metabolism, which could lead to potentially beneficial, novel therapeutic strategies to many patients.

## Figures and Tables

**Table 1:** Mouse primer sequence.

Number	Gene	Legend	Gene bank code	Forward primer (5′ to 3′)	Reverse primer (5′ to 3′)	Product length (bp)	Tm (°C)
1	IL-1*β*	Interleukin 1 beta	16176	TGCCACCTTTTGACAGTGATG	ATGTGCTGCTGCGAGATTTG	136	59.0/59.6
2	IL-6	Interleukin 6	16193	CTGCAAGAGACTTCCATCCAG	AGTGGTATAGACAGGTCTGTTGG	131	58.4/59.2
3	TNF-*α*	Tumor necrosis factor	21926	CAGGCGGTGCCTATGTCTC	CGATCACCCCGAAGTTCAGTAG	89	60.2/60.5
4	*β*-Actin	Actin, beta	11461	CTGTCCCTGTATGCCTCTG	ATGTCACGCACGATTTCC	218	56.9/55.8

**Table 2:** Human primer sequence.

Number	Gene	Legend	Gene bank code	Forward primer (5′ to 3′)	Reverse primer (5′ to 3′)	Product length (bp)	Tm (°C)
1	IL-1*β*	Interleukin 1 beta	3553	TCCGACCACCACTACAGCAAGG	GGAGCGTGCAGTTCAGTGATCG	223	64.2/64.0
2	IL-6	Interleukin 6	3569	AGCCACTCACCTCTTCAGAACG	TGCCTCTTTGCTGCTTTCACA	119	62.2/61.0
3	TNF-*α*	Tumor necrosis factor	7124	CAGGCGGTGCTTGTTCCTCAG	CGATGCGGCTGATGGTGTGG	398	63.8/64.1
4	GAPDH	Glyceraldehyde-3-phosphate dehydrogenase	2597	ACAACTTTGGTATCGTGGAAGG	GCCATCACGCCACAGTTTC	101	58.6/59.8
5	p65	RELA proto-oncogene, NF-*κ*B subunit	5970	AGAGGAGCACAGATACCACCAAGAC	AAGCAGAGCCGCACAGCATTC	328	63.8/64.1
6	Nrf2	Nuclear factor erythroid 2 like 2	4780	TGACAATGAGGTTTCTTCGGCTACG	GGAGAGGATGCTGCTGAAGGAATC	385	63.3/63.0
7	PPAR*α*	Peroxisome proliferator-activated receptor alpha	5465	CAAGTGCCTTTCTGTCGGGATGTC	CACCAGCGTCTTCTCAGCCATAC	289	63.9/63.4

## Data Availability

The data used to support the findings of this study are available from the corresponding authors upon request.

## Supplementary Materials

Supplementary Materials Figure S1: (a) HepG2 and LO2 cells were treated with different concentrations of H_2_O_2_ for 3 h; cell viability was measured by CCK-8 assay. (b) Level of GSH in HepG2 cells after treatment with various concentrations of OA. (c, d, e) Relative TNF-*α*, IL-1*β*, and IL-6 expression of HepG2 cells treated with various concentrations of OA. Values are expressed as the mean ± SD from three independent experiments; ^∗∗^*p* < 0.01, ^∗^*p* < 0.05. Bars indicate the standard deviation of the mean. Figure S2: H_2_O_2_ induced the phosphorylation of the AKT/mTOR pathway. (a) Western blot analysis of (p)PI3K/(p)AKT/(p)mTOR protein levels with or without H_2_O_2_ treatment. (b, c, d) Densitometry analysis of (p)PI3K/(p)AKT/(p)mTOR protein levels. ^∗∗^*p* < 0.01, ^∗^*p* < 0.05, compared with the control group. Data are plotted as the mean ± SD from three independent experiments. Bars indicate the standard deviation of the mean. Figure S3: (a, b) relative PA and OA content of mouse liver after surgical procedure. (c, d) Relative PA and OA content of HepG2 cells after being treated with OA. ^∗∗^*p* < 0.01, ^∗^*p* < 0.05, compared with the control group. Data are plotted as the mean ± SD from three independent experiments. Bars indicate the standard deviation of the mean. Figure S4: (a) Western blot analysis of (p)PI3K and LC3-B protein levels after oxidative stress in HepG2 cells with or without OA treatment. Values are expressed as the mean ± SD from three independent experiments; ^∗∗^*p* < 0.01, ^∗^*p* < 0.05. Bars indicate the standard deviation of the mean.
