# Supplementary material for: Oleic Acid Protects against Hepatic Ischemia and Reperfusion Injury in Mice by Inhibiting AKT/mTOR Pathways
Source: Oxid Med Cell Longev. 2019 Dec 13;2019:4842592. doi: 10.1155/2019/4842592 (PMC6930725; doi:10.1155/2019/4842592)

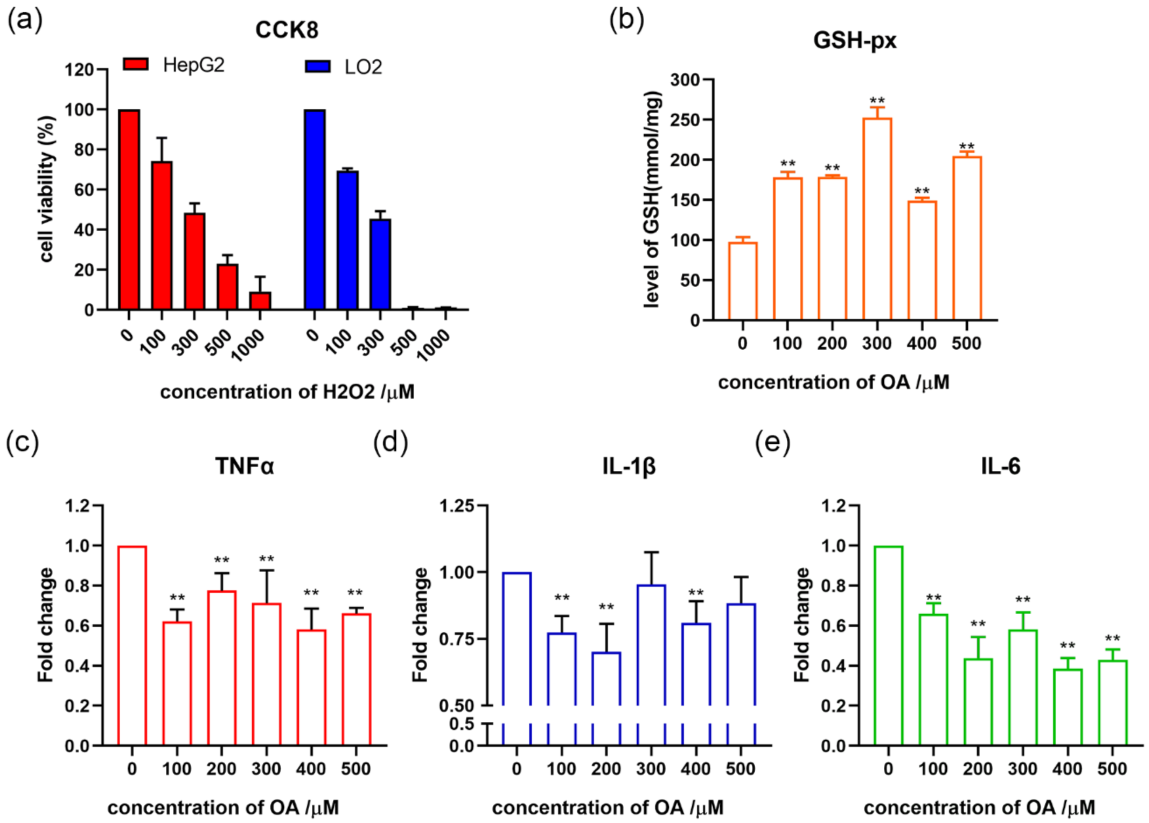

FIGURE S1: (a) HepG2 and LO2 cells were treated with different concentration of H_2O_2 for 3h, cell viability was measured by CCK8 assay. (b) Level of GSH in HepG2 cells after treatment with various concentrations of OA. (c, d, e) Relative TNF-α, IL-1β and IL-6 expression of HepG2 cells treated with various concentrations of OA. Values are expressed as the mean ± s d from three independent experiments. $^{**}P<0.01$, $^{*}P<0.05$. Bars indicate the standard deviation of the mean.

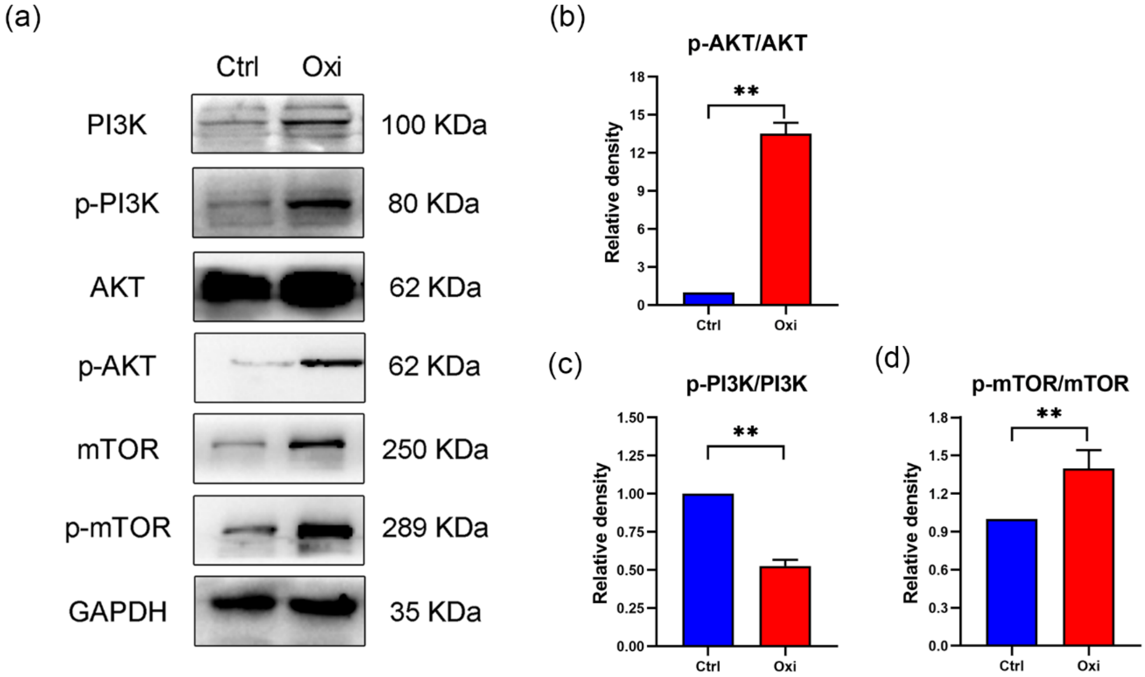

FIGURE S2: H_2O_2 induced the Phosphorylation of the AKT/mTOR pathway. (a) Western blot analysis of (p)PI3K/(p)AKT/(p)mTOR protein levels with or without H_2O_2 treatment. (b, c, d) Densitometry analysis of (p)PI3K/(p)AKT/(p)mTOR protein levels. **P<0.01, *P<0.05, compared with control group. Data are plotted as the mean ± s d from three independent experiments. Bars indicate the standard deviation of the mean.

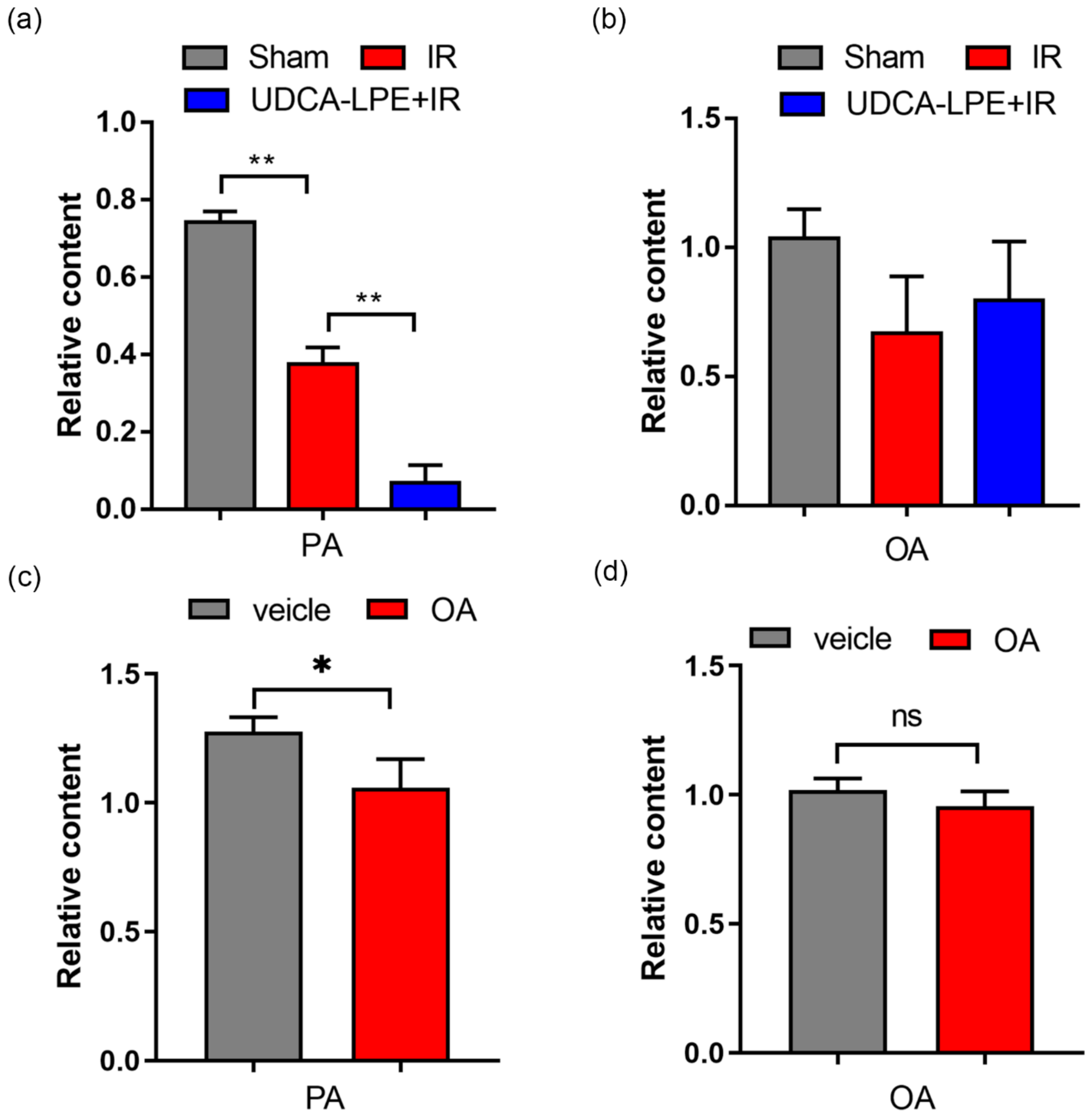

FIGURE S3: (a, b) Relative PA and OA content of mouse liver after surgical procedure. (c, d) Relative PA and OA content of HepG2 cells after treated with OA. **P<0.01, *P<0.05, compared with control group. Data are plotted as the mean ± s d from three independent experiments. Bars indicate the standard deviation of the mean.

(a)

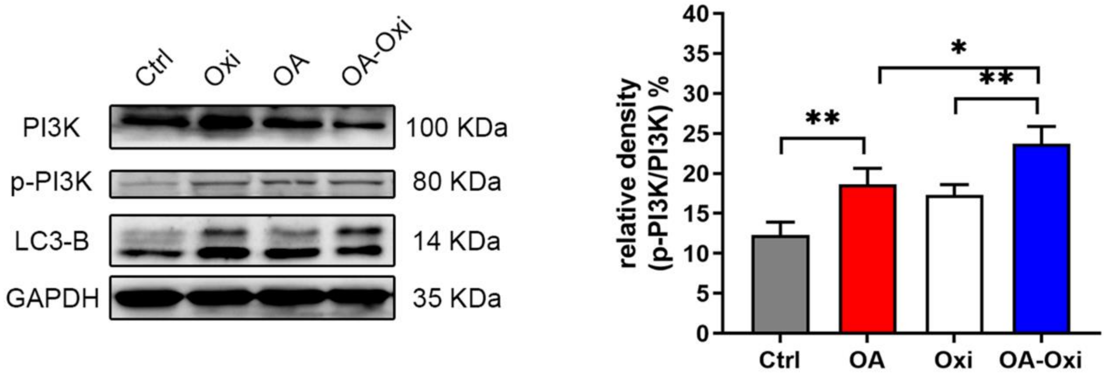

FIGURE S4: (a) Western blot analysis of (p)PI3K and LC3-Ⅱ protein levels after oxidative stress in HepG2 cells with or without OA treatment. Values are expressed as the mean ± s d from three independent experiments. **P<0.01, *P<0.05. Bars indicate the standard deviation of the mean.

Graphical abstract

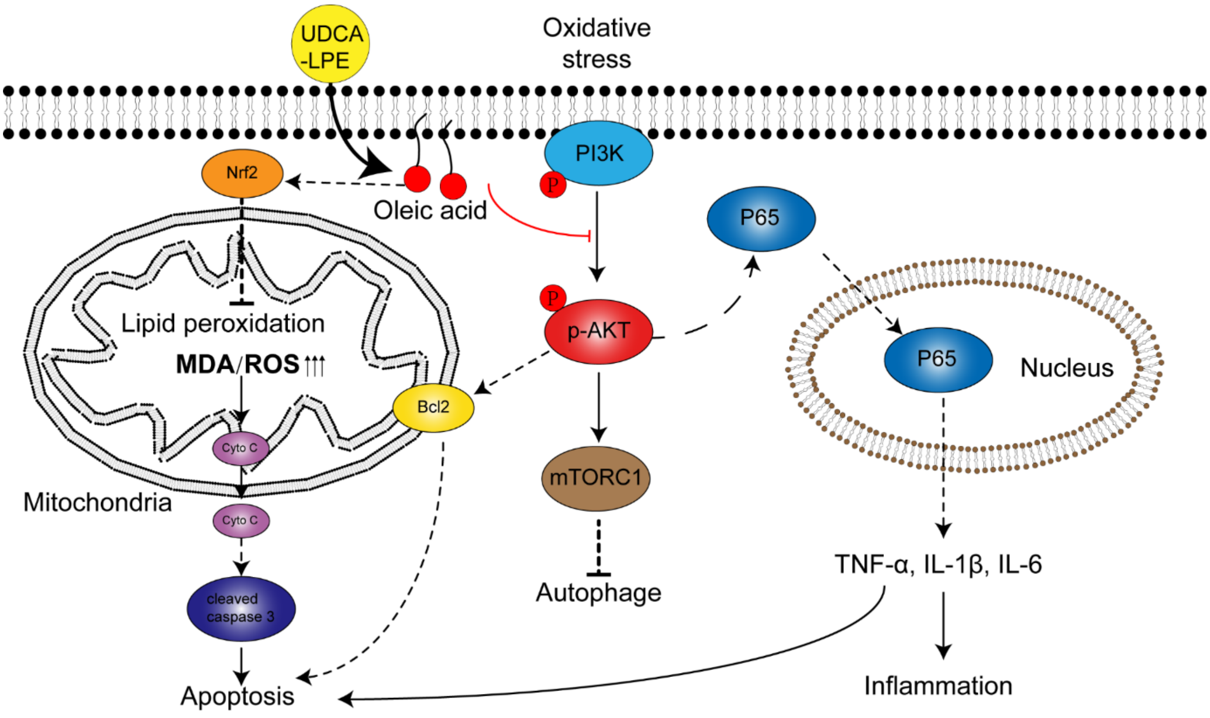

Supplement: Supplementary Materials — Figure S1: (a) HepG2 and LO2 cells were treated with different concentrations of H2O2 for 3 h; cell viability was measured by CCK-8 assay. (b) Level of GSH in HepG2 cells after treatment with various concentrations of OA. (c, d, e) Relative TNF-α, IL-1β, and IL-6 expression of HepG2 cells treated with various concentrations of OA. Values are expressed as the mean ± SD from three independent experiments; ∗∗p < 0.01, ∗p < 0.05. Bars indicate the standard deviation of the mean. Figure S2: H2O2 induced the phosphorylation of the AKT/mTOR pathway. (a) Western blot analysis of (p)PI3K/(p)AKT/(p)mTOR protein levels with or without H2O2 treatment. (b, c, d) Densitometry analysis of (p)PI3K/(p)AKT/(p)mTOR protein levels. ∗∗p < 0.01, ∗p < 0.05, compared with the control group. Data are plotted as the mean ± SD from three independent experiments. Bars indicate the standard deviation of the mean. Figure S3: (a, b) relative PA and OA content of mouse liver after surgical procedure. (c, d) Relative PA and OA content of HepG2 cells after being treated with OA. ∗∗p < 0.01, ∗p < 0.05, compared with the control group. Data are plotted as the mean ± SD from three independent experiments. Bars indicate the standard deviation of the mean. Figure S4: (a) Western blot analysis of (p)PI3K and LC3-B protein levels after oxidative stress in HepG2 cells with or without OA treatment. Values are expressed as the mean ± SD from three independent experiments; ∗∗p < 0.01, ∗p < 0.05. Bars indicate the standard deviation of the mean. [file 4842592.f1.pdf]
